# Willingness to Use Internet-Based Versus Bibliotherapy Interventions in a Representative US Sample: Cross-sectional Survey Study

**DOI:** 10.2196/39508

**Published:** 2022-08-24

**Authors:** Robinson De Jesús-Romero, Akash Wasil, Lorenzo Lorenzo-Luaces

**Affiliations:** 1 Department of Psychological and Brain Sciences Indiana University Bloomington Bloomington, IN United States; 2 Department of Psychology University of Pennsylvania Philadelphia, PA United States

**Keywords:** psychotherapy, digital mental health, digital health, eHealth, adoption, preference, self-help, bibliotherapy, iCBT, CBT, internet-based intervention, self-guided intervention, mental health, print media, cognitive behavioral therapy, digital health intervention, patient education, psychoeducation, health resource, health information, health education, education material

## Abstract

**Background:**

Self-help interventions have the potential to increase access to evidence-based mental health care. Self-help can be delivered via different formats, including print media or digital mental health interventions (DMHIs). However, we do not know which delivery format is more likely to result in higher engagement.

**Objective:**

The aims of this study were to identify if there is a preference for engaging in print media versus DMHIs and whether there are individual differences in relative preferences.

**Methods:**

Participants were 423 adults between the ages of 18 and 82 years (201/423, 47.5% female) recruited on *Prolific* as a nationally representative sample of the US population, including non-Hispanic White (293/423, 69.2%), non-Hispanic Black (52/423, 12%), Asian (31/423, 7%), Hispanic (25/423, 6%), and other individuals (22/423, 5%)*.* We provided individuals with psychoeducation in different self-help formats and measured their willingness to use print media versus DMHIs. We also assessed participants’ demographics, personality, and perception of each format’s availability and helpfulness and used these to predict individual differences in the relative preferences.

**Results:**

Participants reported being more willing to engage with print media than with DMHIs (B=0.41, SE 0.08; t_422_=4.91; *P*<.001; *d*=0.24, 95% CI 0.05-0.43). This preference appeared to be influenced by education level (B=0.22, SE 0.09; t_413_=2.41; *P*=.02; *d*=0.13, 95% CI –0.06 to 0.32), perceived helpfulness (B=0.78, SE 0.06; t_411_=13.66; *P*<.001; *d*=0.46, 95% CI 0.27-0.66), and perceived availability (B=0.20, SE 0.58; t_411_=3.25; *P*=.001; *d*=0.12, 95% CI 0.07-0.30) of the self-help format.

**Conclusions:**

This study suggests an overall preference for print media over DMHIs. Future work should investigate whether receiving mental health treatment via participants’ preferred delivery format can lead to higher engagement.

## Introduction

### Background

Mental health disorders are the leading cause of disability worldwide [1]. However, the demand for mental health services has consistently exceeded the supply, and in recent times, this demand has continued to increase [2,3]. Therefore, innovative delivery of interventions that do not require the presence of a mental health professional may be one way of addressing the supply-demand gap. Cognitive behavioral therapy (CBT), for example, has shown efficacy across a variety of self-help formats [4].

### Self-help Interventions

Self-help can be guided (ie, a self-help intervention with support by a trained professional or paraprofessional) or unguided (ie, self-guided, with no support). Guided self-help has been found to be more effective than unguided self-help [4]. However, unguided self-help has a greater potential for large-scale dissemination [5,6], and it is more effective than control conditions including care as usual or being allocated to a waiting list [5]. Self-help CBT can be delivered in many formats, including digital mental health interventions (DMHIs) that use smartphone apps, web pages, or other web-based formats to deliver the intervention. DMHIs can be highly accessible, with a wide range of resources publicly available on the internet [6]. While DMHIs are a promising way to reduce the public health burden of untreated depression and anxiety, users are currently being inundated with information and options for web-based self-help, most of which are not evidence-based [7]. Additionally, internet access and technical difficulties can be barriers to engaging with DMHIs [8,9].

Self-help interventions can also be delivered through written and print media, which is usually known as bibliotherapy. Meta-analytic reviews suggest that self-help delivered via print media is an effective delivery format [10]. Bibliotherapy is a promising model for disseminating CBT and other empirically supported treatments because it is effective, reasonably cheap, and circumvents the technological barriers associated with internet-based self-help interventions.

Although previous studies have established the efficacy of print media self-help and DMHIs when compared to treatment as usual and other controls for reducing depression [11,12], few studies have explored individuals’ preferences for different delivery formats. Furthermore, even fewer studies have investigated how individuals might differ in their preference to use one format over the other. Individual differences may be especially relevant for understanding engagement with self-help since these interventions tend to suffer from low engagement rates [4]. Thus, it is essential to understand which factors could increase the likelihood of individuals engaging in self-help interventions.

### Treatment Preferences

According to the Theory of Planned Behavior (TPB) [13], attitudes, norms, and perceptions can be used to predict behaviors such as treatment-seeking and engagement. Different studies have tested this theory, confirming a strong relationship among attitudes, intention, and behavior [14]. In psychotherapy, for example, willingness to engage in treatment has been hypothesized to be a proxy for treatment-seeking [15]. Thus, attitudes toward the use of one format over the other could potentially be used to test which type of self-help format users would be more likely to engage with.

Furthermore, it is possible that certain sociodemographic traits could influence the preference for one treatment format over another. For example, younger individuals might be more strongly influenced by attitudes to engage with newer technologies than older individuals, who are influenced more by perceived behavioral control and subjective social norms [16]. In addition, other variables such as education and race could potentially impact the preference for print media versus DMHIs. For example, prior research has suggested that racial and ethnic minorities are less likely than non-Hispanic White individuals to seek and receive treatment owing to barriers such as stigma, health care engagement, and policies [17]. Thus, self-help might potentially be an alternative for individuals who are less likely to engage in traditional care, though it is unclear whether one self-help format would be preferred to the other. The broader literature on treatment engagement points to the role of variables including the presence of distress, maladaptive emotion regulation strategies, and personality as being predictors of engagement in treatment.

### This Study

Our first objective was to explore the relative attitudes of individuals toward the use of self-help in print media (ie, bibliotherapy) versus an internet-based format (ie, DMHIs). We explored this question by providing individuals with psychoeducation on self-help and measuring their willingness to use bibliotherapy versus internet-based self-help, the perceived availability of the format, and its perceived helpfulness. Our second objective was to identify whether demographic and attitudinal variables predicted willingness to use one intervention over the other. We included variables related to treatment outcomes and engagement such as psychological distress, personality, and self-efficacy. We also added a COVID-19–related question to assess whether the pandemic influenced the outcomes.

## Methods

### Recruitment

Participants were adults over 18 years of age recruited via *Prolific* (N=423)—a web-based participant panel shown to be an effective way of collecting high-quality data from diverse participants for research purposes [18]. The sample was stratified by *Prolific* to be representative of the US population in terms of age, sex assigned at birth, and race/ethnicity according to the US Census Bureau. The study was advertised as being about “preferences for mental health treatments” and hosted on the Qualtrics website.

### Ethical Considerations

The study was approved by the internal review board of the University of Pennsylvania (843424). All participants had to consent to the study by reading the informed consent form and clicking that they consented to participate before commencing the survey. The informed consent form included information on the purpose of the study, future use of the data, possible risks, and researchers’ contact information. Participants were compensated with US $5 for their time.

### Measures

#### Treatment Attitudes

Participants were presented with basic information regarding different treatment alternatives, including print media and internet-based self-help. Print media was described as “self-help books designed by psychologists and mental health professionals that include information and exercises designed to help people learn skills that improve their mental health or well-being.” DMHIs were described as “websites, computer programs, or smartphone apps designed by psychologists and mental health professionals. These tools include information and exercises designed to help people learn skills that improve their mental health or well-being. In unguided online self-help programs and smartphone apps, individuals learn content from a website or an app on their own.”

After reading about each treatment option, the survey asked about their willingness to try the intervention (ie, “If I were seeking support for my mental health or well-being, I would be willing to try this option”), perceived efficacy (ie, “I believe this option could be helpful for people looking to improve their mental health or well-being”), and perceived availability of the intervention (ie, “I believe this option is available and accessible for people looking to improve their mental health or well-being”). Responses to the questions about willingness, efficacy, and availability were rated on a 7-point Likert scale (1=strongly disagree, 7=strongly agree) [19].

#### Psychological Distress

We measured psychological distress using the Kessler Psychological Distress Scale (K6) [20]. The K6 is a 6-item scale assessing internalizing distress (ie, nervousness and depression) by asking participants to rate on a 4-point scale how often they have experienced negative affect symptoms over the past month (0=none of the time, 4=all of the time). Scores ranged from 0 to 24, with higher scores indicating higher distress. Specifically, scores of 6 may indicate mild distress, and scores of 13 may indicate more severe distress. The K6 has been validated and demonstrated to have criterion validity [21] and was an internally consistent measure of internalizing distress in a nationally representative sample (α=.89) [20,22].

#### Expressive Suppression

We measured expressive suppression using the suppression subscale of the Emotion Regulation Questionnaire (ERQ-SUP) [23]. This is a 6-item subscale assessing participants’ habitual use of expressive suppression by asking participants how much they agree with specific statements (eg, “I keep my emotions to myself”) on a 7-point scale (1=strongly disagree, 7=strongly agree). Scores ranged from 4 to 28, with higher scores representing higher habitual use of suppression. The ERQ-SUP has been validated and shown to have criterion validity [24] and internal consistency (α=.76-.96) [25].

#### Personality

We assessed the Big-Five personality traits (ie, neuroticism, extraversion, agreeableness, conscientiousness, and openness) using the Ten Item Personality Inventory (TIPI) [26]. This is a 10-item scale assessing personality traits with 5 bipolar factors representing extraversion, agreeableness, conscientiousness, emotional stability, and openness to experience. The measure contains 2 descriptors for each pole of all 5 personality dimensions. Each of these is rated using a 7-point scale (1=disagree strongly, 7=agree strongly). After reverse coding, the mean for each of the 5 personality dimensions were used as subscales. The TIPI has been validated and demonstrated to have adequate factor structure, convergent validity [27], and internal validity (α=.40-.73) [26].

#### Self-efficacy

We also measured self-efficacy using the General Self-Efficacy Scale (GSF) [28]. This is a 10-item scale assessing participants’ general sense of perceived self-efficacy by asking participants how much each specific statement feels true (eg, “I can usually handle whatever comes my way”) on a 4-point scale (1=not at all true, 7=exactly true). Scores ranged from 10 to 40, with higher scores representing higher perceived self-efficacy. The GSF has been validated and shown to have criterion and internal validity (α=.75-.91) [29].

#### Effect of the COVID-19 Pandemic

The survey also asked participants how the COVID-19 pandemic has affected their willingness to consider mental health treatment options that are not delivered in person. Participants could choose between “more likely,” “less likely,” and “no change” regarding their willingness to engage in other forms of treatment.

### Statistical Analyses

All analyses were conducted using R [30] with the RStudio graphical user interface [31]. First, we present descriptive statistics to characterize the sample, including mean (SD) values for continuous variables and n (%) values for categorical variables. Our first objective was to determine whether participants, on average, were more willing to use print media or DMHIs. To explore this question, we conducted a paired sample samples *t* test, which tests if the within-person difference in preferences was significantly different from 0. With a sample size of 423 participants, the study was powered at 80% to detect minor differences (ie, *d*=0.14) at *P*<.05. We also report differences in the perceived efficacy and availability of print media and DMHIs. Our second objective was to determine whether baseline demographic and clinical variables affected the willingness to use print media versus internet-based self-help. Because there is very little theoretical or empirical work on preferences for print media versus DMHIs, we used a machine learning algorithm to select variables that could serve as individual differences in willingness to use print media versus DMHIs. Specifically, we used a model-based recursive partitioning with random forests to help us identify subgroups of observation with different parameters to the basic model, which describes the overall within-person difference in preference for print media versus internet-based self-help. In other words, this procedure allowed us to identify potential predictors of the difference in willingness to use print media versus DMHIs [32]. A significant moderator of this relationship would imply that different subgroups of individuals differ in the extent to which they prefer print media versus DMHIs. Model-based recursive partitioning using random forests explores potential moderators by bootstrapping to identify the most influential moderators. We tested 1000 bootstrap samples. We used model-based recursive partitioning for variable selection because it has been successfully used in studies of individual differences in psychological interventions. The method is able to assess a large number of variables, test nonlinear relationships, and ultimately corresponds well with our research question (ie, whether the overall difference in preference is moderated by third variables).

The variables that were selected as candidate moderators were then entered into a linear regression predicting differences in willingness. To assess whether other attitudinal variables contributed to differences in willingness, we added differences in perceived availability and perceived helpfulness to the regression model with demographics, personality, and clinical variables.

## Results

### Demographics

Participants were 423 adults between the ages of 18 and 82 years (201/423, 47.5% female). The sample was representative of the US population, except that we undersampled American Indian or Alaskan Native and Pacific Islander individuals, who were not present in the sample (Table 1).

**Table 1:** Demographic characteristics of a nationally representative sample of 423 Prolific users.

Variables			Values
Age (years), mean (SD)			45 (16)
**Gender identity, n (%)**			
	Male	213 (50.4)	
	Female	201 (47.5)	
	Gender-queer or gender non-conforming	9 (2.1)	
**Sexual orientation, n (%)**			
	Heterosexual	361 (85.3)	
	Not heterosexual+	62 (14.6)	
**Race, n (%)**			
	Non-Hispanic White	293 (69.3)	
	Non-Hispanic Black	52 (12.3)	
	Asian	31 (7.3)	
	Hispanic	25 (5.9)	
	Other	22 (5.2)	
**Education, n (%)**			
	Postgraduate	96 (22.7)	
	Bachelor’s degree	163 (38.5)	
	High school or less	164 (38.8)	
Yearly income (US $), mean (SD)			71,000 (49,000)
Expressive suppression (Emotion Regulation Questionnaire)			2.85 (1.41)
Psychological distress (Kessler Psychological Distress Scale score), mean (SD)			6.77 (5.77)
Self-efficacy (General Self-Efficacy Scale score), mean (SD)			2.13 (.56)
Agreeableness (Ten Item Personality Inventory [TIPI] score), mean (SD)			5.30 (1.28)
Conscientiousness (TIPI score), mean (SD)			5.26 (1.40)
Extraversion (TIPI score), mean (SD)			3.38 (1.64)
Neuroticism (TIPI score), mean (SD)			4.68 (1.70)
Openness (TIPI score), mean (SD)			5.13 (1.27)
Perceived helpfulness of digital mental health interventions, mean (SD)			4.73 (1.33)
Perceived availability of digital mental health interventions, mean (SD)			5.67 (1.27)
Perceived helpfulness of print media, mean (SD)			5.11 (1.21)
Perceived availability of print media, mean (SD)			6.03 (1.07)

### Overall Preference

On average, participants reported higher willingness to use print media (mean 4.77, SD 1.82) rather than DMHIs (mean 4.37, SD 1.81). Comparing the within-person difference in willingness to use print media versus internet-based self-help revealed a significant but small preference for print media (B=0.41, SE 0.08; *t_422_*=4.91; *P*<.001; *d*=0.24, 95% CI 0.05-0.43). Most participants reported being more willing to use print media (178/423, 42.1%) than DMHIs or no preference (159/423, 37.5%). Few preferred to use DMHIs than print media (86/423, 20%).

### Predictors of Willingness

**Figure 1:**
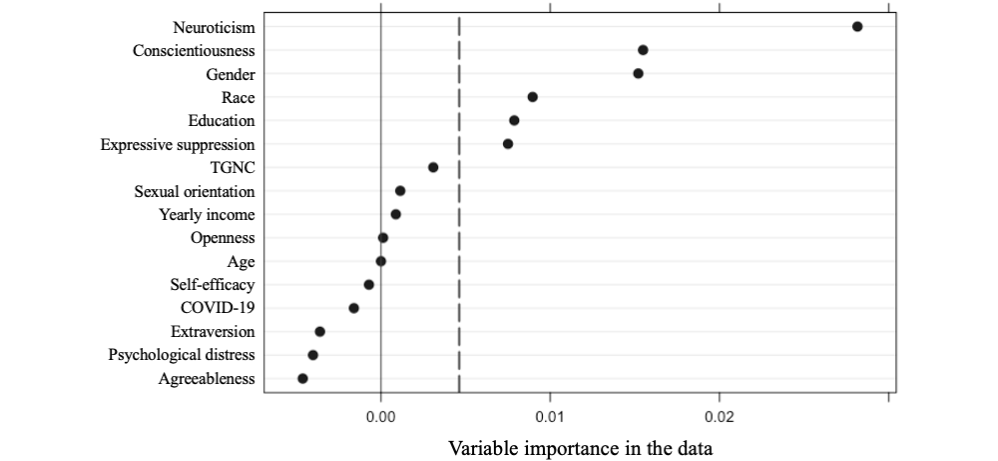
Variable importance plot for variables predicting willingness to engage in print media rather than internet-based self-help, representing mean decreases in accuracy when removing each variable. TGNC: transgender or gender non-conforming.

We explored whether baseline variables moderated the preference for print media over DMHIs using model-based recursive partitioning via random forests. The variable importance plot ranked neuroticism, conscientiousness, expressive suppression, and participants’ gender, race, and education as the top predictive variables (Figure 1).

The variables identified by the MobForest algorithm were then included in a multiple regression as predictors of willingness to use print media over DMHIs. Including these variables yielded a significant overall regression model (*R*^2^=0.43, *P*=.03). This model suggested that greater education is associated with a higher willingness to use print media over DMHIs (B=0.22, SE 0.09; t_413_=2.41; *P*=.02; *d*=0.13, 95% CI –0.06 to 0.32). Across all but the lowest education levels (ie, high school or less), participants preferred print media DMHIs, and this preference was strongest among the most educated participants. Furthermore, identifying as Black (vs Non-Hispanic White) suggested a greater preference for print media to internet-based self-help (B=0.49, SE 0.25; t_413_=1.92; *P*=.06; *d*=0.29, 95% CI 0.09-0.48).

### Attitudes as Predictors of Willingness

We added attitudinal variables, namely differences in the perception of the helpfulness and availability of print media versus DMHIs to the linear model. When adding these variables to the regression model, race and education were no longer significantly associated with willingness to use print media versus internet-based self-help (*R*^2^=0.40, *P*<.001). Perceived differences in helpfulness were strongly associated with the willingness to use print media versus DMHIs (B=0.78, SE 0.06; t_411_=13.66; *P*<.001; *d*=0.46, 95% CI 0.27-0.66). The perceived availability of print media versus DMHIs also affected willingness to use, though these effects were smaller (B=0.20, SE 0.58; t_411_=3.25; *P*=.001; *d*=0.12, 95% CI 0.07-0.30).

## Discussion

### Principal Findings

Our main findings indicate that most participants were more willing to use print media rather than DMHIs. This preference appeared to be influenced by education level, perceived availability, and perceived helpfulness of the DMHI. Specifically, higher perceived helpfulness had the most substantial effect on participants’ willingness to use print media rather than DMHIs. Furthermore, a higher education level was associated with a stronger preference for print media than for DMHIs.

### Sociodemographic Predictors of Willingness to Engage

Previous research has suggested an association between lower education and a higher risk of symptom deterioration when engaging in DMHIs [33]. Thus, regardless of the format, making materials more understandable and engaging for individuals with lower education might be an important avenue for research. It is also important to note that age was not associated with the preference for print media over internet-based self-help. Previous research has suggested that younger individuals may be more likely to engage in newer technologies based on attitudes [16], such as perceived helpfulness or availability. Race was also associated with preferences. Specifically, Black individuals reported a stronger preference for print media than for DMHIs. However, this association was weak and requires further research.

### Attitudes

The perceived helpfulness and availability of the self-help intervention format seem to be useful for understanding a participant’s willingness to engage in self-help. Our finding alludes to a stronger preference toward print media because participants perceive it as potentially more helpful. These data could be used to improve efforts to engage individuals in treatment and personalize treatment allocation on the basis of individual preferences. In other words, willingness to use internet-based self-help could be optimized by using the information on the efficacy of internet-based self-help versus that of print media. According to the TPB, willingness to engage in treatment can be used as a proxy for treatment-seeking [34,35]. Thus, the relationship between attitudes and willingness to engage in self-help interventions could have potential implications for future efforts to increase use and engagement in evidence-based self-help interventions. Additionally, it is possible that engagement with DMHIs could be improved if individuals had print media to support their use of DMHIs.

### Limitations, Strengths, and Future Directions

Before interpreting the results of this study, several limitations are worth noting. First, although *Prolific* data appear to be of higher quality than those obtained from college student samples and from other web-based panels, the possibility of a selection bias affecting our results cannot be ruled out. Indeed, existing data suggest that individuals in web-based panels tend to be more depressed than the average adult in the United States. It must be noted that while this means that our sample is different from a representative US sample, it is not clear whether this would bias our findings. Our primary question is with regard to the differences between print media and DMHIs. We may nonetheless expect that people in a web-based panel may have stronger preferences for DMHIs than for print media given that they are already engaged with online tools such as *Prolific*.

Additionally, we measured self-reported willingness to use different self-help formats and not actual engagement with the content. Although willingness has been found to predict engagement, it is not a perfect predictor of actual behavior. Furthermore, we did not measure other aspects of the TPB including subjective norms and perceived behavioral control. Finally, although the sample was broadly representative of the US population, it undersampled American Indian or Alaskan Native and Pacific Islander individuals. Nevertheless, several strengths are worth considering. First, the study was powered to detect minor differences that may have practical implications, for example, for large-scale dissemination of self-help resources. Additionally, we measured a variety of individual differences and used machine learning to identify factors that could be germane to treatment engagement.

One logical future direction is to test whether willingness to engage in treatment can predict actual engagement in treatment and how perceived helpfulness influences this relationship. Therefore, future studies could test whether providing education on the efficacy of interventions could increase participants’ willingness to use them. Another alternative would be to test whether allocating patients to their preferred format could lower dropout rates, as our study suggested individual differences in preferences for the different interventions [36]. While DMHIs have received attention over the past couple of years, our results suggest that individuals may be more interested in print media. The combination of DMHIs with print media could be a potential avenue to explore to increase engagement with self-help materials.

### Conclusions

Self-help interventions have the potential to improve access to mental health resources. Although there is great excitement for DMHIs, it is essential to remember that this is not the only available format. This study revealed an overall preference for print media over internet-based self-help, which seems to be related to the perceived helpfulness of the format. These findings indicate possible future targets for interventions to increase treatment-seeking and reduce dropout rates. These findings are especially important for self-help interventions that suffer from a high dropout rate [5]. Therefore, to optimize the usability of self-help interventions, we need more studies to confirm the association among self-help delivery formats, attitudes, and engagement in the interventions.

## Abbreviations

CBT: cognitive behavioral therapy
DMHIs: digital mental health interventions
ERQ-SUP: Emotion Regulation Questionnaire
GSF: General Self-Efficacy Scale
K6: Kessler Psychological Distress Scale
TIPI: Ten Item Personality Inventory
TPB: Theory of Planned Behavior
